# Knowledge, Attitudes, and Intentions towards Human Papillomavirus Vaccination among Nursing Students in Spain

**DOI:** 10.3390/ijerph16224507

**Published:** 2019-11-15

**Authors:** Sara Villanueva, Diego Gabriel Mosteiro-Miguéns, Eva María Domínguez-Martís, David López-Ares, Silvia Novío

**Affiliations:** 1 School of Nursing, University of Santiago de Compostela, 15782 A Coruña, Spain; saritavipe@hotmail.com; 2 Galician Public Health Care Service, University Hospital Complex of Santiago de Compostela (CHUS), 15706 A Coruña, Spain; diegomoste@gmail.com; 3 Galician Public Health Care Service, Health Care Centre of Ordes, 15680 A Coruña, Spain; eva.dominguez2@hotmail.com; 4 Galician Public Health Care Service, University Hospital Complex of A Coruña (CHUAC), 15006 A Coruña, Spain; dlopares@gmail.com; 5 Department of Psiquiatry, Radiology, Public Health, Nursing and Medicine, University of Santiago de Compostela, 15782 A Coruña, Spain

**Keywords:** attitude, intention, knowledge, nursing, Papillomaviridae, vaccination

## Abstract

Human papillomavirus (HPV), which is linked to specific types of cancer, can be prevented by vaccination. This study aimed to determine the knowledge and attitudes of nursing students about HPV and its vaccine as well as their intentions towards personal vaccination. A total of 536 Spanish nursing students were invited to complete the Spanish version of the questionnaire “Knowledge, attitudes and intentions towards HPV”. Overall, 367 surveys were completed (68.4% response rate). Data analysis included the calculation of three scores: the knowledge score, categorized into low (<33%), moderate (33%–66%), and good knowledge (>66%); the attitude score, sorted into positive (<2.5), neutral (2.5–3.5), and negative attitude (>3.5); and the intention score, categorized into not favorable (<4), neutral (4–7), and favorable intention (>7). Knowledge about HPV and its vaccine was moderate (54.34 ± 0.9%), and the attitude towards vaccination was positive (2.34 ± 0.03). The intention towards personal vaccination increased significantly after completing the questionnaire (before: 4.14 ± 0.27, after: 6.02 ± 0.28; *p* < 0.001). The present study highlights the need of training future nurses about HPV and its vaccine, considering the important role it plays in the prevention of sexually transmitted diseases.

## 1. Introduction

Human Papillomavirus (HPV) is the most common sexually transmitted infection in both sexes. It is estimated that most men and women will contract the infection at some point in their lives [1]. HPV is a group of more than 200 viruses of which one-quarter are transmitted directly by sexual, oral, vaginal, or anal contact, even if the carrier is asymptomatic [2]. According to their oncological pathogenesis, these viruses are classified by risk type into two groups: low and high risk. HPV 6 and 11, the most common low-risk types of HPV, cause genital warts, whilst the high-risk HPV types, among which HPV 16 and 18 stand out, are related to cancer, specifically, to cervical, oropharyngeal, anal, vaginal, vulvar, and penile cancers [3]. The most recent data for Spain, extracted from the Cancer Spanish Registry REDECAN, estimate that a total of 1987 and 8486 (6049 men and 2437 women) new cases of cervical and oropharyngeal cancer will occur, respectively, in the year 2019 [4].

There actually exist three types of vaccine against HPV (Cervarix ^®^, Gardasil ^®^, and Gardasil 9 ^®^). They are highly effective for the prevention of HPV [5], particularly when they are administered before the beginning of any sexual activity [6]. Hence, by the end of 2017, 80 countries were recommending its administration [7]. In Spain, although each Autonomous Community is responsible for organizing its health care system, the vaccine against HPV is included in all immunization calendars. Concretely, as mostly recommended in other European countries [8], it is only offered to girls at around 12 years of age (0- and 6-month schedule) [8,9]. However, the global coverage of vaccination on a national level is lower than 80% [10].

This lack of coverage as well as the absence of these vaccines in the Spanish vaccination calendar for boys could be related to a lack of preventive education or misinformation about them, which even exists in the healthcare field [11,12]. In this sense, in a study carried out with medical students, it was noticed that the 49.5% of the students did not know that the vaccine protects against other types of cancer, apart from cervical cancer, and 21% were unaware that it can also be administered to men [11]. Likewise, among the factors outlined by students with medical training as reasons for not having received the vaccine were the lack of knowledge about the availability of the vaccine and the mistrust in the protection that this could offer [12].

Besides physicians, nurses play a vital role in the prevention of diseases through vaccination, so they are cornerstones in health promotion and protection. To date, among the few studies which have been carried out with nursing students to evaluate the knowledge, awareness, and/or beliefs regarding HPV infection and vaccination [13,14,15,16], only one included European students, specifically, Italian ones [14]. However, there are significant differences between Italian and Spanish vaccination calendars and nursing degree programs. Thus, the objective of this study was to determine the knowledge and attitudes about HPV and its vaccine as well as the intentions with regard to vaccination among a sample of Spanish nursing students.

## 2. Materials and Methods

### 2.1. Design

An observational cross-sectional descriptive study was carried out.

### 2.2. Setting and Participants

All the nursing students of the University of Santiago de Compostela (USC, Galicia, Spain), one of the three public universities in Galicia, were invited to participate in the study. The investigation included students of both sexes, aged 18 years or older, enrolled in the nursing degree in the 2017–2018 academic year, who voluntarily agreed to participate. On the contrary, participants who had not studied only in a Galician University were excluded.

The size of the study population was 536 at the time of the research. Keeping the expected frequency of all variables at 50%, the desirable sample size using a 95% confidence interval was determined to be 317. However, allowing a non-response rate of 15% and after rounding off, the final desired sample size was determined to be 365.

### 2.3. Translation and Transcultural Adaptation

Translation–backtranslation was the methodology used to make the semantic and cultural adaptation of the questionnaire “Knowledge, attitudes, and intentions towards HPV vaccination (KAI-VPH)”, following the guidelines of Beaton et al. [17] and Sperber et al. [18]. Two Spanish translations of the original version of the KAI-VPH questionnaire were made by two bilingual people, who were outside the research team and had a wide experience in the diagnosis and treatment of HPV infection. In order to verify the adequacy of the translations, these were revised by the investigator team to obtain a unified version of the questionnaire in Spanish (Table S1). Then, the backtranslation process was conducted. Following the same procedure, the unified Spanish version was again translated into English by two people who did not know the original version of the questionnaire. Conceptual and semantic equivalence was analyzed for each item. Finally, a pilot study was carried out with 10 students, who did not participate in the final study, in order to evaluate the clarity and ease of understanding of the items. None of the 39 items in the questionnaire were considered inappropriate for the Spanish context, and only minimal changes were made following the pilot study.

### 2.4. Data Collection

The information was obtained from the Spanish version (Table S1) of the original English questionnaire developed by Dany et al. [19].

The questionnaire consists of 39 items structured into 4 sections. The first section includes sociodemographic data and personal information (habits, sexual experience, etc.). The second section consists of 16 dichotomous items (true or false) and assesses the level of knowledge of HPV infection and its vaccination. After this section, students are given information about HPV and its vaccine before they can proceed to the third section, which assesses their attitudes towards HPV vaccination using 9 statements with a 5-point Likert scale for each (from 1: strongly agree to 5: strongly disagree). Finally, the fourth section is a 10-point scale that determines the intention of the participant to get vaccinated after going through all of the sections of the questionnaire (from 1: not at all likely to 10: extremely likely).

In order to assess the reproducibility of the second section of the questionnaire, 15 students who did not participate in the final study filled in the first two sections of the questionnaire twice at an interval of 15 days between them.

The questionnaires were anonymous and self-completed between February and April of 2018. Students were free to omit any questions that they did not want to answer. In order to get high participation levels, two methods were used to distribute the questionnaires: (i) Distribution of the questionnaires in person, which were filled in during the break between classes and left on a desk once they were filled in; (ii) Mailing of the questionnaires via the application “Google Surveys”.

### 2.5. Ethical and Legal Consideration

The use of the KAI-HPV questionnaire was authorized by the author of the original instrument, Dr. Dany. The study was performed with the approval of the Faculty of Nursing, University of Santiago de Compostela. Likewise, after explaining the procedure and the objective of the investigation, we obtained the consent of the students, whose participation was completely voluntary. Pursuant to the Declaration of Helsinki and Data Protection Act (Organic Law 3/2018), data confidentiality was guaranteed at all times.

### 2.6. Statistical Analysis

The results were presented as the mean ± standard deviation (SD) or the number and percentage, as appropriate. Based on the participants’ responses, three scores were calculated; knowledge, attitude, and intention scores, as previously described by Dany et al. [19]. Knowledge scores, ranging from 0% to 100% correct answers, were categorized as low (<33%), moderate (33%–66%), and good knowledge (>66%); attitude scores, ranging from 1 (most positive) to 5 (most negative), were sorted into positive (<2.5), neutral (2.5–3.5), and negative attitude (>3.5); and intention scores, ranging from 0 (lower intention) to 10 (higher intention), were categorized into not favorable (<4), neutral (4–7), and favorable intention (>7).

Each score was compared across the demographic characteristics by using ANOVA, Mann–Whitney U, or student T-tests. Furthermore, bivariate logistic regression was used to determine the relationships among the demographic characteristics and the knowledge, attitude, and intention scores. The mean values of intention scores before and after the study were compared using paired sample t-tests.

In relation to the psychometric properties of the questionnaire (second section), the Cronbach α coefficient was calculated, and the test–retest reproducibility was studied by the intraclass correlation coefficient.

A *p*-value less than 0.05 was considered significant throughout the study. The software IBM SPSS Statistics (version 24, IBM, Madrid, Spain) was used for the statistical processing of the data.

## 3. Results

### 3.1. Knowledge, Attitudes, and Intentions towards the HPV Vaccine

#### 3.1.1. Description of the Sample

A total of 367 students of the Degree of Nursing from the USC decided to participate in the study (response rate of 68.4%): 118 first-year students, 106 second-year students, 113 third-year students, and 30 fourth-year students. The remaining students did not answer the questionnaire online or they were absent from class on the day the questionnaire was administered. All the students who filled in the questionnaires answered all the questions.

Table 1 shows the demographic characteristics of the participants. Most were non-smoking middle-class women who did not consume alcohol or drank occasionally, had sexual experience, and were vaccinated against HPV.

#### 3.1.2. Knowledge of HPV Infection and Vaccination

In general, knowledge of HPV infection was insufficient (Table 2). Although a large number of students knew about the association of HPV with cervical cancer and genital warts, as well as the main routes of infection and the conditions in which HPV can be transmitted, their ignorance about the Papanicolau test, the treatment of the HPV infection, and the groups at risk should be noted. On the other hand, it needs to be stressed that the knowledge of the HPV vaccine would have been good if it were not for the students’ ignorance of the immunization guidelines and cost (Table 2 and Table 3).

Statistically, the mean knowledge score was 54.34% ± 0.9%, which reflects a moderate level of knowledge. This score was significantly higher for students aged ≥21 years, in the last years of their degree, with high economic status and previous sexual experience, who knew about their HPV vaccination status and about the existence of the vaccine—mainly through information that they had received from their general practitioner or gynecologist (Table 3). However, when bivariate logistic regression was undertaken with the variable “good knowledge” set as the dependent variable, the results showed that knowledge scores >66% were only more likely among students in the last year of their degree (adjusted OR = 6.10, 95% CI = 1.26–9.58).

#### 3.1.3. Attitude towards HPV Vaccination

In general, the majority of participants said that they would recommend the vaccine to their friends or thought that the HPV vaccination should be recommended by gynecologists (Table 4) taking into account (i) the potential risk of transmission; (ii) the seriousness of the diseases associated with HPV infection; (iii) the risk/benefit ratio; and (iv) the efficacy of the vaccination.

Statistically, the mean attitude score was 2.34 ± 0.03, which reflects an attitude with a slight positive trend towards vaccination. In general, female students, participants who drank alcohol, and those who were vaccinated showed a more positive attitude than male students, participants who did not drink alcohol, and those who were not vaccinated, respectively (Table 3). When bivariate logistic regression was undertaken with the variable “positive attitude” set as the dependent variable, the results showed that attitude scores <2.5 were only more likely among female students (adjusted OR = 2.96, 95% CI = 1.72–5.11).

#### 3.1.4. Intentions of Receiving HPV Vaccination

The intention of receiving the HPV vaccine improved significantly after the completion of the questionnaire and the administration of information about HPV (Table 5), with this being significantly more favorable between first-year middle-class students who were between the ages of 19 to 20 and who had unhealthy lifestyles. When bivariate logistic regression was undertaken with the variable “favorable intention” set as the dependent variable, the results showed that intention scores >7 were more likely among students who were smokers (adjusted OR_previous_ = 7.08, 95% CI = 3.49–9.57; adjusted OR_after_ = 4.77, 95% CI = 1.21–8.87).

### 3.2. Psychometric Validation

The validation of the questionnaire involved analyzing its reliability (internal consistency and reproducibility) and validity. The Cronbach α value for the knowledge domain was 0.76, indicating acceptable internal consistency. Regarding the test–retest analysis (n = 15), the intraclass correlation coefficient for the 15 students who completed the questionnaire twice was excellent: 0.898 (95% confidence interval 0.697–0.966). On the other hand, content validity was demonstrated, since the questionnaire was based on expert consensus.

## 4. Discussion

In the present study, the majority of the nursing students had insufficient knowledge about the etiology, the groups at risk, the diagnosis, and the treatment of HPV infection. These results confirm the necessity to introduce changes in the current curriculum of the Spanish nursing degree in order to increase students’ understanding about HPV and promote positive attitudes towards vaccination. To our knowledge, this is the first study carried out in Spain to evaluate the level of knowledge and attitudes of nursing students towards HPV and its vaccine as well as their intentions with respect to the vaccination.

Nursing staff play an important role in the development of preventive measures and in promoting vaccination among the general population, who often do not know the vaccines exist or may be hesitant about receiving them due to misconceptions that exist in our society [20]. This places healthcare staff in a privileged position regarding patient care and management. However, it is necessary for them to have excellent training, which should be evaluated periodically, in order to detect gaps in their knowledge which, at times, are prompted by changes in healthcare practices. Regarding this aspect, it is worth mentioning that a modification in the Spain’s vaccination schedule against HPV was introduced in 2018 (two-dose schedule instead of three-dose schedule), an aspect that was widely unknown by the nursing students (~90%).

When the knowledge as well as the attitudes towards HPV infection and its vaccine were examined in previous studies, which were carried out among student nurses [13,14,15,16] or even nurses [21,22,23,24,25,26,27,28], the results were generally worse and more negative, respectively, than the ones obtained in present study. However, the comparisons with all of them probably do not make sense when we take into account that (i) all of them, except three studies which included American [26], Italian [14], and Canadian [22] populations, were carried out in underdeveloped or developing countries (Uganda, Turkey, Nigeria, Thailand, Tanzania, India) and the one with American nurses dates back to more than 20 years ago and (ii) the level of training stipulated in underdeveloped or developing countries is usually inferior to the training stipulated in developed countries [29]. Due to these reasons and because we focused our efforts on discovering educational needs during undergraduate studies of nursing, the results of the present study should be only compared to those of the study carried out by Pelullo et al. [14].

Both the Italian students [14] and the students in the present study did not have problems with identifying the main type of HPV-related cancer (84.7%); however, both cohorts of students, especially the Italian one, had more difficulty in recognizing the connection between HPV infection and genital warts (29.9% Italian students and 61.3% Spanish students). Likewise, most of the students from both studies (more than 80%) were aware that HPV is a sexually transmitted disease, although 58.3% of the Italian students did not know that sexual intercourse at an early age was a risk factor for HPV infection. As expected, the knowledge of the students improved over the years, probably due to the training that they received during their degree, in the same way that other studies have noted an improvement according to the years of professional experience [30] or after receiving training sessions [31]. In light of these results, the differences in the nursing degree curriculum between Italy and Spain (3 versus 4 years) do not seem to affect the training achieved on this subject. However, we can neither rule out the possibility that the Italian students had received more specific training in vaccination than the Spanish ones, given that Pelullo et al. [14] did not specify whether the nursing students included in the study were doing a General Nursing Degree or a Pediatric one (the latter is non-existent in Spain).

An answer that caused us great concern was the high percentage of Spanish students who failed to recognize that HPV infection could be contracted by men just as easily as by women (only 50% versus 85.6% for the Italians). The greatest lack of knowledge of the Spanish students could be related to differences in the target population regarding the vaccine against HPV. The National Health Care System in Italy authorizes the administration of the vaccine for free to both boys and girls, whereas in Spain, it is only given to girls for free, and the parents of boys who are interested in receiving the vaccine have to purchase it from the chemist. This is the reason why we thought it would be interesting to include a question in the questionnaire about the cost of the vaccine, as its high price could be a restricting factor for many low-income families [13], and furthermore, it would help to explain why the levels of vaccine coverage are lower in males than in females (in the current study, none of the men were vaccinated versus the 91.8% of the women who said they were vaccinated). Taking into account that the implication of HPV in diseases which may affect both men and women has been proved, primary prevention of HPV-related diseases should be done in both genders.

The percentage of vaccinated Italian students was clearly inferior to the percentage registered in our study (23.9% versus 73%, respectively). However, when students were asked about their intention to be vaccinated against HPV, the Italian students were more receptive (65.3%) than the Spanish ones at first. However, it should be noted that the intention of being vaccinated for the Spanish students improved after providing them with information about the virus and the vaccine (before: 4.14 and after: 6.02). This stresses the efficiency of educational measures not only to increase students’ level of knowledge but also to modify their attitude. Likewise, the majority of Spanish (81.7%) and Italian (91.7%) students were willing to recommend the HPV vaccine to friends or other people because they thought it was useful and safe. Nevertheless, what really struck us was that although more than 80% of Spanish students correctly identified the routes of transmission and associated this infection with cervical cancer, approximately 30% recognized that they always or sometimes had sexual relationships without the use of contraception. This could have been attributed to the lack of knowledge that transmission can occur even when the patients have no symptoms (70% were unaware of this) or to the self-perceived low risk of contracting the HPV infection (only 36% of the students thought themselves to be at risk).

Our study has several limitations. The main limitation is related to the students’ participation, which decreased as students advanced from year to year and could be associated with the rate of class attendance (higher in the first year of the degree; fourth-year students are an exception as they are in Practicums all year round). Second, as students filled in the questionnaires themselves, there may have been some self-report bias. Third, another limitation of the study would include the absence of information regarding private university students’ knowledge and attitudes of nursing students about HPV and its vaccine, as there are no private universities in Galicia. Because of this limitation, we cannot generalize our results to nursing students with other characteristics (e.g., students who are socioeconomically advantaged) a priori. Fourth, bearing in mind the prevalence of high-risk HPV serotypes in Spanish women [32,33] and considering that not all of them are included in the currently available vaccine programs, it would have been interesting to determine the knowledge of nursing students about the level of protection offered by these vaccines; thus, there is a misunderstanding that the HPV vaccination is an alternative to cervical screening programs rather than a complementary strategy for cancer prevention [34,35]. Likewise, the false sense of protection of these vaccines could lead to a higher prevalence of risky lifestyles or sexual habits, such as a lower use of condoms [33,35], which is of great significance, especially for younger women [32].

## 5. Conclusions

In light of the results of the current study, it is verified that future nurses need training about HPV and its vaccine due to the important roles they play in the prevention of sexually transmitted diseases, some of them related to the occurrence of cancer. This finding emphasizes the need for making a change in the approach to vaccine education of the current nursing curriculum. It would be necessary to replace its current purely theoretical approach with an eminently practical one. Students should work on clinical cases reflecting real-world conditions where they have (i) to decide the route of administration of the vaccine; (ii) to know vaccine-related immediate or late side effects and their management; (iii) to know what vaccines can be co-administered.

## Figures and Tables

**Table 1:** Baseline characteristics of the study’s participants.

	N (%)	Mean ± SD
Number of participants	367 (68.4)	
Age (years):		21.6 ± 3.80
19−20	168 (45.78)	
≥21	199 (54.22)	
Sex:		
Female	292 (79.6)	
Male	75 (20.4)	
Program:		
First year	118 (32.1)	
Second year	106 (28.9)	
Third year	113 (30.8)	
Fourth year	30 (8.2)	
Religion:		
Christians	203 (55.3)	
Muslim	1 (0.3)	
Atheism	163 (44.4)	
Economic status:		
Low	2 (0.5)	
Middle	341 (92.9)	
High	24 (6.5)	
Smoking status:		
Smoker	47 (12.8)	6.31 ± 5.61 ^a^
Non-smoker	320 (87.2)	
Drinking status:		
Drinks alcohol	169 (46.0)	3.38 ± 3.06 ^b^
Does not drink alcohol	198 (54.0)	
Sexual history:		
No sexual experience	44 (12.0)	
Sexual experience(s) always with the use of contraception	209 (57.1)	
Sexual experience(s) sometimes with the use of contraception	98 (26.8)	
Sexual experience(s) always without the use of contraception	15 (4.1)	
HPV vaccination status:		
Vaccinated	268 (73.0)	
Not vaccinated	64 (17.4)	
DK/NO	35 (9.6)	
Awareness of HPV vaccine:		
Heard about the vaccine before	364 (99.2)	
Never heard about the vaccine before	3 (0.8)	

Abbreviations: DK/NO: do not know/no opinion; HPV: human papillomavirus; SD: standard deviation. ^a^ Number of cigarettes per day; ^b^ Number of glasses per week.

**Table 2:** Knowledge of HPV infection and vaccination.

Item (Correct Answer)	Correct N (%)	Incorrect N (%)	DK/NO N (%)
The type of cancer highly associated with HPV infection is uterine cervical cancer (true)	311 (84.7)	15 (4.1)	41 (11.2)
Human papillomavirus can cause herpes (false)	30 (8.2)	198 (54)	139 (37.9)
Human papillomavirus can lead to genital warts (growths on the skin of the genitals) (true)	225 (61.3)	6 (1.6)	136 (37.1)
HPV can be transmitted through vaginal, anal, and oral sex as well as genital to genital contact (true)	294 (80.1)	13 (3.5)	60 (16.3)
In most cases, HPV-infected women do not show symptoms (true)	247 (67.3)	24 (6.5)	96 (26.2)
All HPV infections are caused by the same type of virus (false)	166 (45.2)	50 (13.6)	151 (41.1)
HPV-positive pregnant women can pass the virus to their babies (false)	21 (5.7)	176 (48.0)	170 (46.3)
Only females can be infected with HPV and show symptoms (false)	189 (51.5)	83 (22.6)	95 (25.9)
HPV can be transmitted from a carrier to his/her partner only if the carrier shows symptoms (false)	255 (69.5)	14 (3.8)	98 (26.7)
A normal pap smear implies that the woman is free of HPV (false)	75 (20.4)	78 (21.3)	214 (58.3)
There is no current cure or therapy for HPV infection (true)	93 (25.3)	116 (31.6)	158 (43.1)
HPV vaccines have the same effect whether the female takes it before or after being infected with HPV (false)	267 (72.8)	10 (2.7)	90 (24.5)
HPV vaccine is best taken before starting to have sexual activities (true)	300 (81.7)	16 (4.4)	51 (13.9)
HPV vaccine can only be taken after the age of 18 years (false)	320 (87.2)	8 (2.2)	39 (10.6)
HPV vaccination is taken as three injections over a period of six months (false)	40 (10.9)	251 (68.4)	76 (20.7)
HPV vaccination costs around 30 euros (false)	147 (40.1)	19 (5.2)	201 (54.8)

**Table 3:** Students’ knowledge and attitude scores stratified by their characteristics.

	Knowledge Score (Mean ± SD)	*p*	Attitude Score (Mean ± SD)	*p*
All participants	54.34 ± 0.9		2.34 ± 0.03	
Age				
19−20 years	47.58 ± 1.19	<0.001	2.31 ± 0.03	0.327
≥21 years	60.05 ± 1.17		2.36 ± 0.03	
Sex				
Male	51.91 ± 2.36	0.172	2.55 ± 0.51	<0.001
Female	54.96 ± 0.95		2.28 ± 0.48	
Program				
First year	45.65 ± 1.56	0.001	2.28 ± 0.04	0.106
Second year	54.83 ± 1.39 *,**		2.44 ± 0.04	
Third year	62.33 ± 1.51 *		2.30 ± 0.04	
Fourth year	56.66 ± 1.51 *		2.35 ± 0.10	
Religion				
Christians	55.20 ± 1.12	0.363	2.36 ± 0.03	0.327
Atheism	53.56 ± 1.43		2.31 ± 0.04	
Economic status				
Middle	53.68 ± 0.92	0.007	2.33 ± 0.02	0.346
High	63.54 ± 1.49		2.43 ± 0.05	
Smoking status				
No	54.21 ± 0.98	0.720	2.35 ± 0.02	0.158
Yes	55.18 ± 2.17		2.24 ± 0.07	
Drinking status				
No	55.14 ±1.19	0.335	2.39 ± 0.03	0.036
Yes	53.40 ±1.36		2.28 ± 0.03	
Sexual history				
No sexual experience	49.57 ± 2.12 ***	0.006	2.36 ± 0.06	0.842
Sexual experience(s) always without the use of contraception	65.83 ± 1.04		2.38 ± 0.09	
Sexual experience(s) always with the use of contraception	53.43 ± 1.20 ***		2.32 ± 0.03	
Sexual experience(s) sometimes with the use of contraception	56.63 ± 1.82		2.37 ± 0.05	
HPV vaccination status				
Not vaccinated	57.91 ± 2.20 ****	<0.001	2.57 ± 0.06 *****	<0.001
Vaccinated	55.34 ± 0.97 ****		2.26 ± 0.02	
DK/NO	40.80 ± 2.37		2.49 ± 0.07	
Awareness of HPV vaccine				
Never heard about the vaccine before	27.08 ± 6.27	0.006	2.63 ± 0.22	0.319
Heard about the vaccine before	54.56 ± 0.89		2.33 ± 0.02	

Significant differences (*p* < 0.05) with respect to students: * of first year of degree studies; ** of third year of degree studies; *** who had sexual experience(s) always without the use of contraception (e.g., condoms); **** who did not know or did not appear to know if they had been vaccinated; ***** who had been vaccinated.

**Table 4:** Attitudes towards HPV infection and its vaccine.

	Strongly Agree N (%)	Agree N (%)	Neutral N (%)	Disagree N (%)	Strongly Disagree N (%)
Based on my lifestyle, I believe that I am susceptible to the HPV infection and must get the vaccine.	55 (15.0)	77 (21.0)	114 (31.1)	79 (21.5)	42 (11.4)
Based on the general sexual practice among Spanish people, I believe that students in Spain have a good chance of contracting HPV and, therefore, all college students should receive the HPV vaccine	104 (28.3)	153 (41.7)	81 (22.1)	26 (7.1)	3 (0.8)
I believe that contracting HPV virus is serious and life-threatening	149 (40.7)	156 (42.6)	43 (11.7)	16 (4.4)	2 (0.5)
I believe that the current HPV vaccine is capable of preventing the occurrence of cervical cancer	63 (17.2)	181 (49.3)	99 (27.1)	20 (5.5)	2 (0.5)
I believe that the price of the vaccine is affordable, given the benefits it offers	27 (7.4)	54 (14.7)	78 (21.3)	129 (35.1)	79 (21.5)
I believe that the side effects of the vaccine are reasonable and will not deter me from taking the vaccine	103 (28.1)	137 (37.3)	88 (24.0)	34 (9.3)	5 (1.4)
I believe that the HPV vaccine is different from other marketed vaccines produced by pharmaceutical companies with the prime purpose of accumulating profit	21 (5.7)	68 (18.5)	184 (50.1)	81 (22.1)	13 (3.5)
I believe that all gynecologists should recommend the vaccine to their patients, whether or not they come from conservative families	190 (51.8)	118 (32.2)	50 (13.6)	9 (2.5)	0 (0)
I would recommend this vaccine to my college friends whether or not they come from conservative families	191 (52.0)	109 (29.7)	59 (16.1)	7 (1.9)	1 (0.3)

**Table 5:** Intention score to obtain the HPV vaccine before and after the questionnaire stratified by students’ characteristics.

	Intention Score Before Survey (Mean ± SD)	*p*	Intention Score After Survey (Mean ± SD)	*p*
All participants	4.14 ± 0.27		6.02 ± 0.28	<0.001
Age				
19−20 years	4.89 ± 0.37	0.009	6.78 ± 0.36	0.007
≥21 years	3.45 ± 0.37		5.26 ± 0.40	
Sex				
Male	4.01 ± 0.30	0.561	5.97 ± 0.31	0.085
Female	4.39 ± 0.60		5.92 ± 0.65	
Program				
First year	4.74 ± 0.45 *	0.031	6.22 ± 0.39	0.008
Second year	4.67 ± 0.54 *		6.69 ± 0.53 *	
Third year	2.96 ± 0.46		4.33 ± 0.54	
Fourth year	3.18 ± 0.74		6.91 ± 1.00	
Religion				
Christians	4.17 ±0.39	0.820	5.90 ± 0.38	0.832
Atheism	4.04 ±0.38		6.02 ± 0.41	
Economic status				
Middle	4.29 ± 0.28	0.029	6.26 ± 0.27	<0.001
High	2.22 ± 0.79		2.56 ± 0.81	
Smoking status				
No	3.81 ±0.28	0.025	5.71 ± 0.30	0.065
Yes	5.39 ± 0.71		7.06 ± 0.66	
Drinking status				
No	3.63 ± 0.34	0.068	5.25 ± 0.39	0.005
Yes	4.63 ± 0.42		6.80 ± 0.36	
Experience sexual				
No sexual experience	4.86 ± 1.01	0.685	5.86 ± 0.50	0.697
Sexual experience(s) always without the use of contraception	4.00 ± 0.68		5.50 ± 0.34	
Sexual experience(s) always with the use of contraception	3.83 ± 0.35		5.74 ± 0.39	
Sexual experience(s) sometimes with the use of contraception	4.42 ± 0.56		6.45 ± 0.54	
Awareness of HPV vaccine				
Never heard about the vaccine before	3.00 ± 1.00	0.563	4.50 ± 0.50	0.459
Heard about the vaccine before	4.13 ± 0.28		5.99 ± 0.28	

* Significant differences (*p* < 0.05) with respect to students of third year of degree studies.

## Supplementary Materials

The following are available online at https://www.mdpi.com/1660-4601/16/22/4507/s1, Table S1 Spanish version of the questionnaire “Knowledge, attitudes and intentions towards human papilloma virus (HPV) vaccine”. Adapted from Dany et al. (2015).
