# Supplementary material for: Knowledge, Attitudes, and Intentions towards Human Papillomavirus Vaccination among Nursing Students in Spain
Source: Int J Environ Res Public Health. 2019 Nov 15;16(22):4507. doi: 10.3390/ijerph16224507 (PMC6888169; doi:10.3390/ijerph16224507)
Supplement: Supplementary file 1 [file ijerph-16-04507-s001.pdf]

Table S1. Spanish version of the questionnaire "knowledge , attitudes and intentions towards human papiloma virus (HPV) vaccine. Adapted from Dany et al. (2015).

<table>
<tr><th colspan="12">Sección 1: Información general

Por favor, responda las siguientes preguntas personales no identificables.</th></tr>
<tr><td>1</td><td>Año de nacimiento</td><td>— — — —</td><td>2</td><td colspan="3">Facultad:</td><td>3</td><td colspan="4">Sexo:</td></tr>
<tr><td>4</td><td>Nivel de estudios</td><td>Estudiante de primer año</td><td colspan="2">Estudiante de segundo año</td><td colspan="2">Estudiante de tercer año</td><td colspan="2">Estudiante de cuarto año</td><td colspan="3">Graduado</td></tr>
<tr><td>5</td><td>Religión</td><td>Cristiana</td><td colspan="2">Musulmana</td><td colspan="2">Ninguna</td><td colspan="5">Otras ……………………….. (Especifique cuál)</td></tr>
<tr><td>6</td><td colspan="2">Situación económica</td><td colspan="2">Alta</td><td colspan="2">Media</td><td colspan="5">Baja</td></tr>
<tr><td>7</td><td colspan="2">Hábito de fumar</td><td colspan="2">No fumador</td><td colspan="7">Fumador
¿Cuántos cigarrillos al día?.................................</td></tr>
<tr><td>8</td><td colspan="2">Estado de consumo de alcohol</td><td colspan="2">No bebedor</td><td colspan="7">Bebedor
¿Cuántos vasos a la semana?..............................</td></tr>
<tr><td>9</td><td colspan="2">Historia sexual</td><td colspan="9">Sin experiencia sexual
Experiencia(s) sexual (es) siempre sin el uso de contracepción (p.ej. preservativo)
Experiencia(s) sexual(es) siempre con el uso de contracepción (p.ej. preservativo)
Experiencia(s) sexual(es), a veces, con el uso de contracepción (p.ej. preservativo)</td></tr>
<tr><td>10</td><td colspan="2">Ha sido vacunado antes con la vacuna del Virus del Papiloma Humano (VPH)?
(Si su respuesta es Sí, pase a la pegunta 12)</td><td colspan="3">Sí</td><td colspan="2">No</td><td colspan="4">NS/NC</td></tr>
<tr><td>11</td><td colspan="2">En una escala del 1-10: siendo 1 lo menos probable y 10 lo más probable, ¿tiene pensado ponerse la vacuna del Virus del Papiloma Humano (VPH)?</td><td>1</td><td>2</td><td>3</td><td>4</td><td>5</td><td>6</td><td>7</td><td>8</td><td>9</td><td>10</td></tr>
<tr><td>12</td><td colspan="2">Antes de este estudio, ¿había oído hablar sobre la vacuna del Virus del Papiloma Humano (VPH)?
(Si su respuesta es No, pase a la sección 2)</td><td colspan="5">Sí</td><td colspan="5">No</td></tr>
</table>

13	**¿Dónde ha escuchado hablar de la vacuna? (Marque todas las posibles)**	Medios de comunicación<br>Internet<br>Médico de familia o ginecólogo<br>Conferencias de la Universidad o profesores<br>Familia o amigos<br>Otros, por favor, especifique ____________________

Sección 2: Conocimiento sobre el Virus del Papiloma Humano (VPH) y su vacuna.

Esta sección está diseñada para evaluar su conocimiento sobre el Virus del Papiloma Humano y su vacuna. Para cada una de las siguientes afirmaciones, escoja la opción con la que esté de acuerdo. Si no sabe la respuesta, simplemente elija la opción "No sé".

**El tipo de cáncer altamente asociado con la infección por VPH es:**	Cáncer de ovarios	Cáncer de mama	Cáncer cérvicouterino	No sé
**El VPH puede causar herpes.**	Verdadero	Falso	No sé	
**El VPH puede dar lugar a verrugas genitales (crecimientos en la piel de los genitales).**	Verdadero	Falso	No sé	
**El VPH puede ser transmitido mediante sexo vaginal, anal y oral así como por el contacto genital con genital.**	Verdadero	Falso	No sé	
**En muchos casos, las mujeres infectadas con el VPH no muestran síntomas.**	Verdadero	Falso	No sé	
**Todas las infecciones por VPH son causadas por el mismo virus.**	Verdadero	Falso	No sé	
**Mujeres embarazadas con VPH positivo pueden trasmitírselo a su bebé.**	Verdadero	Falso	No sé	
**Sólo las mujeres pueden ser infectadas por VPH y mostrar síntomas.**	Verdadero	Falso	No sé	
**El VPH puede transmitirse del portador a su pareja sólo si éste muestra síntomas.**	Verdadero	Falso	No sé	
**La prueba de Papanicolau normal implica que la mujer está libre de VPH.**	Verdadero	Falso	No sé	
**No existe una cura o terapia actual para la infección por VPH.**	Verdadero	Falso	No sé	
**Las vacunas del VPH tienen el mismo efecto si la mujer es vacunada antes o después de haberse infectado por el VPH.**	Verdadero	Falso	No sé	
**La vacuna del VPH es mejor ponerla antes de comenzar a tener relaciones sexuales.**	Verdadero	Falso	No sé	
**La vacuna del VPH sólo se puede poner después de los 18 años.**	Verdadero	Falso	No sé	
**La vacunación del VPH son tres inyecciones en un período de seis meses.**	Verdadero	Falso	No sé	
**La vacunación cuesta alrededor de 30 euros.**	Verdadero	Falso	No sé	

Necesita saber esta información sobre el VPH y su vacuna antes de continuar:
Los Virus del Papiloma Humano son un grupo de virus humanos capaces de causar varios tipos de infecciones y enfermedades. Se transmiten principalmente a través de las relaciones sexuales. Entre ellos están los virus de alto riesgo de VPH (principalmente VPH 16 y 18) cuya infección está altamente relacionada con el cáncer cérvicouterino, un tipo muy grave de cáncer.
Dos vacunas contra el VPH han sido diseñadas para reducir el riesgo de infección de VPH por transmisión sexual: Gardasil y Cervarix. En el último año se desarrolló otra vacuna, Gardasil 9. A las mujeres de 11 años o más se les recomienda vacunarse. La vacuna también puede administrarse en hombres.
En Galicia, la vacuna consistía en tres inyecciones en un período de seis meses pero, a partir del 2018, se implantó la pauta de dos inyecciones en seis meses. Cada inyección cuesta alrededor de 150 euros (Gardasil) y 120 euros (Cervarix).
La vacuna es más efectiva como medida preventiva antes de contraer la infección viral en lugar de recibir la vacuna después de contraer el virus.
Los efectos secundarios comunes de la vacuna incluyen: dolor e hinchazón en el lugar de la inyección, dolor de cabeza, dolor muscular, fatiga, náuseas, vómitos, diarrea, fiebre y dolor abdominal. Los efectos secundarios poco comunes incluyen mareos e infección del tracto respiratorio superior.

**Sección 3: Actitudes hacia la vacunación contra el Virus del Papiloma Humano (VPH)**<br><br>**Esta sección está diseñada para evaluar sus actitudes hacia la vacuna contra el VPH después de haber adquirido algunos conocimientos generales sobre el tema.**<br><br>**Elija la opción que refleja su opinión sobre cada una de las siguientes afirmaciones.**					
**De acuerdo con mi estilo de vida, creo que soy susceptible a la infección por VPH y debo vacunarme.**	Totalmente de acuerdo	De acuerdo	Neutral	En desacuerdo	Muy en desacuerdo
**De acuerdo con la práctica sexual general en la población española, creo que los estudiantes universitarios tienen bastantes posibilidades de contraer el VPH y, por lo tanto, todos deben recibir la vacuna contra el VPH.**	Totalmente de acuerdo	De acuerdo	Neutral	En desacuerdo	Muy en desacuerdo
**Creo que contraer el virus del VPH es grave y amenaza la vida.**	Totalmente de acuerdo	De acuerdo	Neutral	En desacuerdo	Totalmente en desacuerdo

**Creo que la actual vacuna contra el VPH es capaz de prevenir la aparición de cáncer cérvicouterino**	Totalmente de acuerdo	De acuerdo	Neutral	En desacuerdo	Totalmente en desacuerdo
**Creo que el precio de la vacuna es asequible dados los beneficios que ofrece.**	Totalmente de acuerdo	De acuerdo	Neutral	En desacuerdo	Totalmente en desacuerdo
**Creo que los efectos secundarios de la vacuna son razonables y no me impedirán ponerla.**	Totalmente de acuerdo	De acuerdo	Neutral	En desacuerdo	Totalmente en desacuerdo
**Creo que la vacuna contra el VPH es diferente de otras producidas por compañías farmacéuticas con el objetivo principal de ganar dinero.**	Totalmente de acuerdo	De acuerdo	Neutral	En desacuerdo	Totalmente en desacuerdo
**Creo que todos los ginecólogos deberían recomendar la vacuna a sus pacientes, provengan o no de familias conservadoras.**	Totalmente de acuerdo	De acuerdo	Neutral	En desacuerdo	Totalmente en desacuerdo
**Recomendaría esta vacuna a mis amigos universitarios, provengan o no de familias conservadoras.**	Totalmente de acuerdo	De acuerdo	Neutral	En desacuerdo	Totalmente en desacuerdo

Sección 4:<br>(Responde sólo si NO ha sido vacunado previamente o no sabe)<br><br>Después de hacer la encuesta,<br><br>En una escala del 1–10: siendo 1 menos probable y 10 más probable,<br><br>¿Cuánto está dispuesto a vacunarse ahora con la vacuna contra el VPH?									
1	2	3	4	5	6	7	8	9	10
